# Supervised Machine Learning Techniques for Breeding Value Prediction in Horses: An Example Using Gait Visual Scores

**DOI:** 10.3390/ani14182723

**Published:** 2024-09-20

**Authors:** Fernando Bussiman, Anderson A. C. Alves, Jennifer Richter, Jorge Hidalgo, Renata Veroneze, Tiago Oliveira

**Affiliations:** 1 Animal and Dairy Science Department, University of Georgia, Athens, GA 30602, USA; alvesand@uga.edu (A.A.C.A.); jennifer.richter25@uga.edu (J.R.); jh37900@uga.edu (J.H.); renata.veroneze@uga.edu (R.V.); 2 Animal Science Department, Federal University of Viçosa, Viçosa 36570-900, Brazil; 3 Statistics Department, State University of Paraíba, Campina Grande 58429-500, Brazil; tadolive@servidor.uepb.edu.br

**Keywords:** support vector regression, machine learning, gait prediction, visual scores

## Abstract

**Simple Summary:**

In the artificial intelligence era, much is speculated about the use of machine learning techniques for the most diverse scientific purposes. Machine learning methods are acknowledged to better handle non-linearity and subjectivity than traditional statistical methods. In the horse industry, visual scores are widely used to evaluate gaited horses. This phenotyping strategy is an effective low-cost alternative to more accurate methods. However, since it heavily depends on the person assessing the gait, subjectivity is introduced in the phenotype. Our study evaluated the application of machine learning techniques in the breeding value prediction for visual scores in Brazilian gaited horses. We used a dataset with horses that were measured for at least one of the following gait scores: dissociation, comfort, style, regularity, and development. Traditional methods, such as ordinary least-squares and multiple-trait models, were combined with artificial neural networks and other machine learning regression methods, and each model was evaluated according to its accuracy, bias, and dispersion. Machine learning techniques had accuracy comparable to traditional methods; however, they presented slightly more bias and were over-dispersed. For selection purposes, more studies are needed; however, machine learning techniques are a feasible alternative for unofficial evaluation runs.

**Abstract:**

Gait scores are widely used in the genetic evaluation of horses. However, the nature of such measurement may limit genetic progress since there is subjectivity in phenotypic information. This study aimed to assess the application of machine learning techniques in the prediction of breeding values for five visual gait scores in Campolina horses: dissociation, comfort, style, regularity, and development. The dataset contained over 5000 phenotypic records with 107,951 horses (14 generations) in the pedigree. A fixed model was used to estimate least-square solutions for fixed effects and adjusted phenotypes. Variance components and breeding values (EBV) were obtained via a multiple-trait model (MTM). Adjusted phenotypes and fixed effects solutions were used to train machine learning models (using the EBV from MTM as target variable): artificial neural network (ANN), random forest regression (RFR) and support vector regression (SVR). To validate the models, the linear regression method was used. Accuracy was comparable across all models (but it was slightly higher for ANN). The highest bias was observed for ANN, followed by MTM. Dispersion varied according to the trait; it was higher for ANN and the lowest for MTM. Machine learning is a feasible alternative to EBV prediction; however, this method will be slightly biased and over-dispersed for young animals.

## 1. Introduction

Brazilian gaited horse breeds such as the Campolina are acknowledged to have a natural, smooth four-beat gait called the “marcha” [1], which is further classified into two different gaits according to the proportion of lateral and diagonal supports: marcha picada (MP—when there is a higher proportion of lateral support) and marcha batida (MB—when there is a higher proportion of diagonal support). According to Wanderley et al. [2], MP differs from MB in aspects like speed, range of motion, step frequency, dissociation, and metabolic indicators. Dissociation means that each limb moves in a different rhythm during the horse’s movement [3]. Because of that, each gait (MP or MB) will present different proportions of various support types during locomotion.

Several phenotyping strategies have been proposed in the past few decades to evaluate the gait of gaited horses: kinematics [1,4], body-mounted sensors [5,6], and blood-assessed metabolic profiles [2,7]. Despite those advances, visual kinematic scores are the most common phenotyping strategy used for the genetic evaluation of gaited horses due to its facilitated logistics, reduced cost, speed of phenotyping [8], and relationship to performance in a show arena. Yet, visual scores suffer from subjectivity that is naturally present when different people evaluate the same specific features [8,9,10]. In addition, despite its precision, kinematic video analysis can be time-consuming as it involves a frame-by-frame inspection [1].

There is, however, one factor that all gait phenotypes share in common: they all have a strong environmental influence, which can be attributed (at least partially) either to the appraiser (the person who scores the horse) [8,11] or to the rider [12] and can introduce non-linearity to the observed phenotype. Bussiman et al. [11,13] suggested using the appraiser/technician as a random effect for gait visual scores. They showed that this effect explained more phenotypic variance than the animal additive genetic effect, resulting in a low heritability (0.07–0.16). Thus, selecting for gait is challenging, and genetic gains are limited due to model and phenotyping quality. To overcome these problems, multiple-trait models (MTMs) are typically used to estimate variance components and predict breeding values for gait-related and morphological traits [8,10,11,13,14,15].

Often, visual gait scores show low heritability, which can be due to the subjectivity in the phenotype, large environmental variance, or the technician effect. Because of the reduced heritability, the prediction accuracy is also reduced, lowering the genetic gain. If gait is an economically important trait, new modeling strategies should be assessed with respect to accuracy in order to allow selection for these traits. The prediction accuracy is improved when using MTMs [16,17] mainly because of the information shared or the assessment of genetically correlated traits. In the case of visual gait scores, the genetic correlation with morphological traits, which are widely recorded and usually have higher heritability, can be harnessed using MTMs. Another advantage of MTMs is the reduction in selection bias [18] by using traits measured before and after selection [19]. The MTM’s efficacy depends on the stability of genetic correlations over time, which might change under selection [20,21]. Furthermore, an MTM harnesses more computing power and a proper sample size to allow for efficient estimation [22], and because the number of non-zero elements in mixed model equations increases faster than the number of model effects [23], more calculations are needed for genetic evaluations.

More calculations, however, demand more memory and can increase computing time. If evaluations are run weekly, time can impose a constraint. An alternative to reducing computing costs is data truncation, which is when old or unneeded information is removed [24], or indirect predictions when non-phenotyped animals have their breeding values predicted based only on genomic information [25]. However, data truncation can only be applied in large datasets because removing phenotypes in small datasets could reduce accuracy and increase bias. On the other hand, indirect predictions require the animals to be genotyped. If genomic selection is ongoing, indirect predictions can be used when new animals are coming to the system in between evaluations, and there is a need to compute their breeding values. For non-genotyped new animals, the evaluation is pedigree-based, and the animals’ evaluations would rely only on the parent average since their phenotypes would only be included in the subsequent evaluations.

In this context, one should assess alternative methods that (1) allow the accurate pedigree prediction of animals before they enter official runs and (2) handle non-linear relationships among predictors that can cause higher environmental and technician effects. Supervised machine learning methods handle non-linearity in data by combining different feature attributes and non-linear functions [26]. The term supervised means that the machine learning method “learns” a function by mapping an input to an output based on input–output data pairs [27]. Among these methods, artificial neural networks (ANNs) [28,29], support vector regression (SVR) [27,30], and random forest regression (RFR) [31,32] have been extensively used.

Although these methods have advantages, there are few applications for breeding value prediction. Usually, learning involves using the phenotype to train the models; if that is the case, the uncertainty associated with predictions is a function of heritability: machine learning techniques would perform better for higher heritability than for lower heritability, hence explaining their limited use. This study aimed to investigate the usefulness of ANN, SVR, and RFR to predict breeding values for gait visual scores in Campolina horses using MTM as a benchmark. Additionally, we estimated genetic parameters and genetic trends for all studied traits.

## 2. Materials and Methods

### 2.1. Phenotypes and Phenotyping

According to the 2018 statute [33] of the Brazilian Campolina Breeders Association (ABCCCampolina, www.campolina.org.br accessed on 10 August 2024), after a foal is born, the breeder has to notify the ABCCCampolina about the birth. The foal will be inspected by a technician from the breeders’ association, preferably before weaning. At this moment, a temporary registration is issued. Around 36 months old, at breeders’ will, foals can be inspected again to obtain permanent registration. This second inspection may or may not be conducted by the same technician that inspected the foal at a younger age. As part of this second inspection, animals are measured for various morphometric traits (see Bussiman et al. [11,13]) and five visual gait scores (before and after being ridden) as follows [11,33]:

- Dissociation (Di): This ranges from 0 (no dissociation—trot) to 40 (clear visualization of triple-limb support); it is related to the coordinated movements of thoracic and pelvic limb pairs, with the support and suspension of each pair causing triple-limb support, which guarantees contact with the ground.
- Comfort (C): This varies between 0 (animal with high impacts under saddle carrying the rider uncomfortably) and 60 (animal with no hits under saddle); it is related to the quality of the horse’s movements, with no vertical, lateral, or frontal oscillations and impacts to the rider.
- Style (S): This ranges between 0 (no beauty of movements) and 40 (good balance of limb elevation and elegant movements); it represents the combination of posture, balance and harmony of movements, which need to be elegant and have energy.
- Regularity (R): This ranges from 0 (an animal that changes its gait or loses rhythm) to 30 (an animal that is capable of performing the same gait for long periods of time); this score is associated with the maintenance of the same gait type, conserving itself defined, stable, rhythmic, and with a good cadence.
- Development (De): This varies from 0 (high step frequency) to 30 (low step frequency); this score is related to the capability of the horse to cover long distances with few steps.

Due to the high degree of subjectivity in these scores, the ABCCCampolina provides frequent training for its certified technicians. Hereinafter, the term “technician” refers to the individual who inspects the animal at the time of registration and evaluates its gait. Since the technician appraises the horses’ gait, we use this term interchangeably with “appraiser”. Furthermore, the term “visual” indicates that these scores are based solely on visual inspection, meaning no tools other than the human eye are used.

### 2.2. Available Data

Data from the ABCCCampolina were used in this study. These data contained information on 5891 horses born between 1990 and 2013 with an average age at measurement of 39.65 ± 3.39 months, scored by 46 technicians (Tec), and records for dissociation (Di), comfort (C), style (S), regularity (R), and development (De). For all traits, contemporary groups (CGs) were defined by the concatenation of birth year (24 levels, from 1990 to 2013), sex (2 levels, male and female), and year of registration (24 levels, from 1993 to 2016). Effects to be included in the contemporary groups (CGs) were chosen based on a linear fixed model (i.e., only fixed effects included); then, significant effects were concatenated together to create CGs. This process was carried out individually for each trait, and the same effects ended up being significant for all the traits. This possibly occurred because the traits were measured altogether at the same time. For age as a covariate, its effect was included since horses were measured within a certain range, and by regressing adjusted phenotypes (for CGs) against age, a quadratic equation provided the best fit. The stud was significant for all traits, but since its inclusion in CGs would result in many CG levels with fewer phenotypic records, the stud was fitted as an extra fixed effect. Table 1 presents the descriptive statistics of the dataset.

The related pedigree file had 107,951 horses born between 1951 and 2013 with a total of 14 generations. The average number of foals per stallion was 21.54 (from 4253 stallions), while per mare, it was 3.42 (from 26,760 mares). The average inbreeding coefficient was 2.45% (entire population), and the average relationship (excluding self-relatedness) was 0.02. Additionally, 91,434 horses had both parents known, 177 had only the stallion known, and 23 had only the mare known.

### 2.3. Statistical Analyses

The framework used in this study consists of three main steps: (1) ordinary least squares (OLS), (2) multiple-trait model, and (3) machine learning techniques (MLTs) using the least squares solutions. The third step was further split into three other analyses: (3a) artificial neural network, (3b) support vector regression, and (3c) random forest regression.

For all traits, the following model was implemented:

(1) $$y_{ijk}=\mu +\beta_{1}{age}_{k}+\beta_{2}{age}_{k}^{2}+{CG}_{i}+{Stud}_{j}+e_{ijk}$$

where $y_{ijk}$ represents the phenotypic observations (Di, C, S, R, or De) from the k^th^ horse in the i^th^ CG (contemporary group) and in the j^th^ stud (same as herd); μ is a constant; $\beta_{1}$ and $\beta_{2}$ represent the regression coefficients (linear and quadratic, respectively) of the covariate age at measurement from the kth horse (${age}_{k}$); ${CG}_{I}$ is the cross-classified effect of the i^th^ contemporary group; ${Stud}_{j}$ is the cross-classified effect of the j^th^ stud; and $e_{ijk}$ represent the random residual terms.

### 2.4. Ordinary Least Squares

Under matrix notation, Equation (1) can be written as

(2) y=Xθ+e

where y is the vector of phenotypic observations; X is the incidence matrix for the fixed effects; θ is the solution vector for the fixed effects; and e is the vector of random residuals. Assuming multivariate normality for the residual, the variance of y in Equation (2) is given by

(3) $$Var\left(y\right)=Var\left(e\right)=R=I\sigma_{e}^{2}$$

where y and e are the same as those defined in Equation (2); R is the residual (co)variance matrix with dimensions equal to the number of animals; I is an identity matrix of proper order; and $\sigma_{e}^{2}$ is the residual variance.

The solution of this system of equations is given by

(4) $$\left(X^{T}R^{-1}X\right)\hat{\theta }=\left(X^{-T}R^{-1}\right)y$$

where $X^{T}$ is the transpose matrix of X (defined in Equation (2)); $R^{-1}$ is the inverse of R (defined in Equation (3)); and y is the same as that defined in Equation (2). Because of the number of levels for each fixed effect (Table 1), this model was implemented using the HPMIXED procedure from the software SAS^®^ version 9.4 [34].

### 2.5. Multiple Trait Model

Assuming the same fixed effects as in Equation (1) but including the random animal additive and random technician effects, the model can be written as

(5) $$y_{ijkl}=\mu +\beta_{1}{age}_{k}+\beta_{2}{age}_{k}^{2}+{CG}_{i}+{Stud}_{j}+u_{k}+t_{l}+e_{ijkl}$$

where $y_{ijkl}$ represents the phenotypic observation from the k^th^ horse in the i^th^ CG and in the j^th^ Stud, evaluated by the l^th^ technician; μ, $\beta_{1}{age}_{k}$, $\beta_{2}{age}_{k}^{2}$, ${CG}_{I}$, and ${Stud}_{j}$ are the same as those defined in Equation (1); $u_{k}$ represents the random additive effect of the k^th^ horse; $t_{l}$ represents the random effect of the l^th^ technician; and $e_{ijkl}$ represent the random residual terms.

Under matrix notation, Equation (5) can be written as

(6) $$y=X\theta +Z_{1}u+Z_{2}t+e$$

where y is the vector of phenotypic observations sorted by animal within trait; X is the incidence matrix for the fixed effects; θ is the solution vector for the fixed effects; $Z_{1}$ and $Z_{2}$ are the incidence matrices for the animal additive random effect (u) and technician random effect (t); and e is the vector of random residual terms. Assuming multivariate normality, the distribution of the random effects is given by

(7) $$\left[\begin{array}{c} u\\ t\\ e \end{array}\right]\sim MVN\left\{\left[\begin{array}{c} 0\\ 0\\ 0 \end{array}\right],\left[\begin{array}{ccc} \Sigma_{u}⨂A & 0 & 0\\ 0 & \Sigma_{t}⨂I & 0\\ 0 & 0 & \Sigma_{e}⨂I \end{array}\right]\right\}$$

where u, t, and e are the same as those defined in Equation (6); $\Sigma_{u}$ is the additive genetic (co)variance matrix (5 × 5) among traits; A is the additive relationship matrix; $\Sigma_{t}$ is the technician (co)variance matrix (5 × 5) among traits; $\Sigma_{e}$ is the residual (co)variance matrix (5 × 5) among traits; I is an identity matrix of proper order; and ⨂ denotes the Kronecker product.

For variance component estimation, observations deviating by more than three standard deviations from the phenotypic mean, along with CGs and studs with less than five records (or without variation), were removed (Table 2). Table 3 shows the number of animals measured for each trait. The pedigree file was edited to have only three generations, containing 14,079 horses with an average number of foals per stallion of 7.03 (from 1825 stallions) and 1.76 per mare (from 7281 mares). The average inbreeding was 3.72% (truncated pedigree), and the average relatedness was 0.05 (excluding self-relationships). Additionally, 12,806 horses had both parents known, 29 had only the mare known, and no horse had only the stallion unknown. This was carried out to ensure a proper estimation of the variance components. For the breeding value prediction, the raw dataset and pedigree were used. Unlike variance component estimation, where the goal is to accurately estimate sources of variation, the prediction of genetic merit aims to predict individual animal effects for selection in which using all of the available information helps to obtain the most accurate predictions.

Variance components were estimated via restricted maximum likelihood (REML) using the expectation maximization (EM) algorithm implemented in the software blupf90+ from the BLUPF90 programs family [35]. Breeding values were predicted (once again, using the dataset depicted in Table 1) using the default options of Blupf90+ software.

### 2.6. Machine Learning Techniques

For all MLTs in this study, OLS solutions were used to calculate adjusted phenotypes for each trait as follows:

(8) $$y-X\hat{\theta }=\hat{e}$$

where y and X are the same as those defined in Equation (2); $\hat{\theta }$ is the vector of estimated solutions from Equation (2); and $\hat{e}$ is the vector of adjusted phenotypes. Table 4 presents the descriptive statistics of the adjusted phenotypes along with OLS solutions.

Dummy variables were used to account for the technician effect [36]. We removed the technician with the highest number of records, and a new column was created for each of the remaining technicians; for a given technician, the dummy values were 0 (if the horse was not measured by this technician) or 1 (if the horse was measured by this technician) [37]. Thus, this corresponds to $Z_{2}$ in Equation (6), excluding the column associated with the most frequently assigned technician, with a total of 47 dummy variables. In addition, the first ten eigenvectors of the **A** matrix were used to model the population structure. Dummy variables were created using the function “dummy_cols” from the R package “fastDummies” [38], and eigenvalues of **A** were computed using the function “eigs” from the R package “RSpectra” [39]. Therefore, the “machine learning data” ($X_{MLT}$) were composed by one column for each adjusted phenotype, the corresponding columns for $X\hat{\theta }$ for each trait, dummy variables for the technician, and ten columns due to population structure (eigenvectors of **A**). In all models, the target variable was the EBV obtained in the MTM, and for all further analyses, $X_{MLT}$ was centered so each column had a mean of zero.

#### 2.6.1. Artificial Neural Network

**Figure 1:**
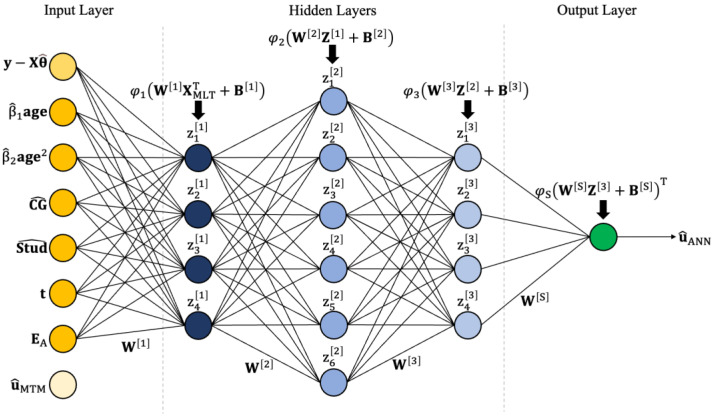
A schematic representation of the trained artificial neural network. In the input layer, each node represents a collection of nodes (one for each trait/effect combination).

The multilayer perceptron was the artificial neural network (ANN) architecture used in this study; it comprises several fully connected layers in a feedforward propagation scheme. Those layers are classified into an input layer, hidden layers, and an output layer. The input layer receives the data (here, $X_{MLT}$), the hidden layers contain the mapping processing units (neurons), and the output layer gives the outcome of the ANN (here, the EBV). By convention, if the number of hidden layers is greater than two, the ANN is considered deep [29]. It was not the objective of this study to evaluate different architectures for the ANN; thus, the implemented topology was composed of three fully connected hidden layers with varying numbers of neurons (Figure 1). Each neuron computes a score, which is mapped (or activated) by a linear or non-linear function (called activation function). Finally, the output layer receives the mapped scores from the last hidden layer to compute the output values (Figure 1).

Let $\hat{u}$ be a vector (n × 1) of EBV (predicted by MTM), with ${\hat{u}}_{i}\sim N\left\{0,\left(1+F_{i}\right){\hat{\sigma }}_{u}^{2}\right\}$, where $F_{i}$ is the inbreeding coefficient of the i^th^ horse and ${\hat{\sigma }}_{u}^{2}$ is the estimated additive genetic variance. Consider $X_{MLT}=\left[\begin{array}{cccc} x_{1} & x_{2} & \cdots & x_{p} \end{array}\right]$ as a matrix (n × p) containing the adjusted phenotypes, OLS solutions, technician dummy variables, and the first ten eigenvectors of **A** for the animals in training (defined later). The number of phenotypic records is n (here, 4324), and the number of features is p (here, 85). The first hidden layer computes the following activated scores:

(9) $$Z^{\left[1\right]}=\varphi_{1}\left(W^{\left[1\right]}X_{MLT}^{T}+B^{\left[1\right]}\right)$$

where $Z^{\left[1\right]}$ is a matrix (h_1_ × n) (where h_1_ is the number of neurons) of activated scores in the first layer; $W^{\left[1\right]}$ is a matrix (h_1_ × p) of weights connecting each neuron to the input layer; $X_{MLT}^{T}$ is the matrix transpose of $X_{MLT}$; $B^{\left[1\right]}$ is a matrix (h_1_ × n) of neuron-specific constants (biases); and $\varphi_{1}\left(x\right)=1/\left(1+e^{-x}\right)$ is the sigmoid activation function.

The second hidden layer performs the following computation:

(10) $$Z^{\left[2\right]}=\varphi_{2}\left(W^{\left[2\right]}Z^{\left[1\right]}+B^{\left[2\right]}\right)$$

where $Z^{\left[2\right]}$ is a matrix (h_2_ × n) of activated scores in the second layer; $W^{\left[2\right]}$ is a matrix (h_2_ × h_1_) of weights connecting each neuron to the first hidden layer; $Z^{\left[1\right]}$ is defined above; $B^{\left[1\right]}$ is a matrix (h_2_ × n) of biases; and $\varphi_{2}\left(x\right)=\left(e^{x}-e^{-x}\right)/\left(e^{x}+e^{-x}\right)$ is the hyperbolic tangent activation function.

The third hidden layer uses the same procedure to calculate the following:

(11) $$Z^{\left[3\right]}=\varphi_{2}\left(W^{\left[3\right]}Z^{\left[2\right]}+B^{\left[3\right]}\right)$$

in which $Z^{\left[3\right]}$ is a matrix (h_3_ × n) of activated scores; $W^{\left[3\right]}$ is a matrix (h_3_ × h_2_) of weights connecting neurons to the previous layer; $Z^{\left[2\right]}$ is defined above; $B^{\left[3\right]}$ is a matrix (h_3_ × n) of biases; and $\varphi_{2}\left(\cdot \right)$ is defined above. Finally, the output layer computes the following:

(12) $${\hat{u}}_{ANN}={\varphi_{3}\left(W^{\left[o\right]}Z^{\left[3\right]}+B^{\left[o\right]}\right)}^{T}$$

where ${\hat{u}}_{ANN}$ is a vector (n × h_o_) of EBV predicted by the ANN; $W^{\left[o\right]}$ is a matrix (h_o_ × h_3_) of weights connecting the neurons in the output layer to the third hidden layer; $Z^{\left[3\right]}$ is defined above; $B^{\left[o\right]}$ is a matrix (h_o_ × n) of biases; and $\varphi_{3}\left(x\right)=x\phi \left(x\right)$ is the gaussian error linear unit activation function (where $\phi \left(x\right)$ represents the cumulative density function of x).

For regression problems, the loss function is generally the mean absolute error (MAE) or the mean squared error (MSE). Both have their advantages and disadvantages. MSE is more sensitive to outliers or significant errors, while MAE gives equal weights to all errors, which can be more robust in the presence of outliers [40]. In this study, the MAE was adopted as the loss function as follows:

(13) $$Loss\left({\hat{u}}_{MTM},{\hat{u}}_{ANN},W\right)=\frac{1}{n}\sum_{i=1}^{n}\left|{\hat{u}}_{{ANN}_{i}}-{\hat{u}}_{{MTM}_{i}}\right|+\lambda {\left\|W\right\|}_{2}^{2}$$

where ${\hat{u}}_{{ANN}_{I}}$ is the EBV predicted by the ANN for the i^th^ horse; ${\hat{u}}_{{MTM}_{I}}$ is the EBV predicted by the MTM for the i^th^ horse; $\left|\cdot \right|$ represents the absolute value; n is the number of horses; $\lambda {\left\|W\right\|}_{2}^{2}$ is L2 regularization to penalize model complexity; W contains the model parameters; ${\left\|\cdot \right\|}_{2}^{2}$ is the squared Euclidean norm; and λ>0 controls the magnitude of the penalty. The learning process involves backpropagating the updated values of W, obtained with some gradient descent method, until $Loss\left({\hat{u}}_{MTM},{\hat{u}}_{ANN},W\right)$ is at its minimum [41].

Once again, it was not the objective of the present study to determine the best architecture of the ANN; therefore, some of the hyperparameters were arbitrarily defined as follows: the optimization algorithm used was the RMSprop due to its ability to minimize the dependence of the learning rate; h_1_ = 4, h_2_ = 6, h_3_ = 4, and h_o_ = 1; epochs = 100; and batch size = n/2. Then, a grid search procedure was performed to find the best learning rate (a) and λ values, testing $\alpha =\left\{\begin{array}{ccc} 0.001 & 0.01 & 0.1 \end{array}\right\}$ and λ from 0.0001 to 1 by increments of 0.0004. The final values were α=0.1 and λ=0.001. The ANN was implemented in the *keras* R package version 2.15.0 [42] using the tensorflow R package version 2.16.0 [43] as a backend.

#### 2.6.2. Support Vector Regression

In a binary classification task, the support vector machine (SVM) algorithm finds an optimum hyperplane such that the decision margin between the two classes is maximized while the misclassification is penalized [27,28,30]. The support vector machine regression (SVR) is an extension of the SVM. However, the main idea is to only use residuals smaller (in absolute value) than a certain constant (ε) called ε-sensitivity [27,30]. This is somehow analogous to the SVM, where the points with correct classification are ignored in the optimization [30]. Finally, the SVR deals with non-linearity in the same way as the SVM, that is, by mapping the input data onto a high-dimensional space where the points are linearly separable [27,28].

Assume $S=\left\{{\hat{u}}_{i},x_{i}\right\}$, i=1,2,⋯,n is a training dataset, with ${\hat{u}}_{i}\sim N\left\{0,\left(1+F_{i}\right){\hat{\sigma }}_{u}^{2}\right\}$ being the EBV predicted by the MTM and $x_{i}$ being a p-dimensional input vector of OLS solutions, technician dummy variables, and the first ten eigenvectors of **A**. By applying Lagrange multipliers, the problem can be represented in terms of support vectors as the following dual optimization problem [44,45]:

(14) $${max}_{a_{i}a_{i}^{*}}\left\{-\varepsilon \sum_{i=1}^{nSV}\left(a_{i}+a_{i}^{*}\right)+\sum_{i=1}^{nSV}{\hat{u}}_{i}\left(a_{i}-a_{i}^{*}\right)-\frac{1}{2}\sum_{i}^{nSV}\sum_{j}^{nSV}\left(a_{i}-a_{i}^{*}\right)\left(a_{i}-a_{i}^{*}\right)k\left(x_{i},x_{j}\right)\right\}$$

subject to the following constraints [30,45]:

(15) $$0\leq a_{i},a_{i}^{*}\leq \frac{1}{\lambda }$$

(16) $$\sum_{i=1}^{nSV}\left(a_{i}-a_{i}^{*}\right)=0$$

(17) $$a_{i}a_{i}^{*}=0$$

in which ε is the maximum residual value (ε-sensitivity); $a_{i}$ are the Lagrange multipliers associated with each observation; ${\hat{u}}_{i}$ is defined above; nSV represents the number of support vectors; $k\left(x_{i},x_{j}\right)=\varphi \left(x_{i}\right)\varphi \left(x_{j}\right)$ is the kernel function; and λ is the L2 regularization parameter. Here, we used the radial basis function, also known as gaussian kernel [27,30]:

(18) $$k\left(x_{i},x_{j}\right)=e^{-\gamma {\left\|x_{i}-x_{j}\right\|}^{2}}$$

in which γ is a user-predefined kernel bandwidth hyperparameter. With larger values of γ, the kernel matrix tends to an identity, hence risking overfit; on the other hand, γ values that are too small reduce the kernel to a constant function, which prevents the learning of nontrivial patterns [27]. The prediction from the SVR of new data (x) is given by

(19) $${\hat{u}}_{{SVR}_{i}}=\hat{f}\left(x\right)=\sum_{i=1}^{nSV}\left(a_{i}-a_{i}^{*}\right)k\left(x_{i},x\right)$$

in which ${\hat{u}}_{{SVR}_{i}}$ is the ith EBV predicted by the SVR, and nSV is the number of support vectors, i.e., the trained data points where $a_{i}>0$. The hyperparameters were chosen based on a combination of grid search with cross-validation. Values varying from 0.0001 to 2 by increments of 0.0124 were tested. The final values of ε, λ, and γ were 0.1, 1, and 0.0125, respectively. The SVR was fit using the e1071 R package version 1.7-14 [46].

#### 2.6.3. Random Forest Regression

Random forest (RF) is a supervised MLT that combines bagging and random split selection [47], building a large collection of decision trees and then averaging out the results [32]. Each tree is built using a splitting criterion (random split) in such a way that the average loss function in the bootstrapped data (bagging) is at its minimum [32]. The idea is that by taking enough bootstrap samples, the prediction variance of a prediction function is reduced [31]. RF regression (RFR) is a type of RF in which the response variable is continuous, and similarly to the classification case, the RFR predicts the outcome by splitting the predictor space [47]. In practical terms, RFR fits a regression tree to each of the many bootstrap samples of the training data and then averages out the prediction [31,32].

Let β be a random vector such that the prediction $\hat{h}\left(x,\beta \right)$ from the tree is a continuous variable and assume the training set ($X_{MLT}$) is independently drawn from the distribution of the response variable (${\hat{u}}_{MTM}$) [47]. As before, we assumed ${\hat{u}}_{MTMi}\sim N\left\{0,\left(1+F_{i}\right){\hat{\sigma }}_{u}^{2}\right\}$, and then the mean squared generalization error is given by the following [47]:

(20) $${E\left[{\hat{u}}_{MTM}-\hat{h}\left(X\right)\right]}^{2}$$

in which $\hat{h}\left(X\right)={\hat{u}}_{RFR}$ represents the EBV predicted by the RFR; ${\hat{u}}_{MTM}$ is the EBV predicted via MTM; and ${E\left[\cdot \right]}^{2}$ represents the square of the expected value (average squared).

Each tree is built by the following algorithm [31,32]:

1. From the training dataset, draw a bootstrap sample of size *n*;
2. Grow a random forest tree ($T_{b}$) with specific splitting criterion by the following loop:
  - Draw random *m* variables out of the initial *p* variables;
  - Pick the best variable (or split point) out of *m*;
  - Split the node into two new nodes;
  - Loop until the minimum node size (*n_min_*) is reached.
3. Loop to 1. until *k* trees are grown;
4. Output the forest.

Finally, the prediction from new data points ($x_{i}$) is given by

(21) $${\hat{u}}_{{RFR}_{i}}=\frac{1}{k}\sum_{i=1}^{k}T_{b}\left(x_{i}\right)$$

where k is the number of trees, and $T_{b}\left(x_{i}\right)$ is the prediction from one single tree. It was not the objective of this study to determine the best k, which was fixed at 200 trees. A grid search procedure was used to find the best combination of hyperparameters, testing values from 1 to 10 for minimum node size (*n_min_*) and $\sqrt{p}$, 0.1p, 0.3p, and 0.5p (p is the number of predictor variables in the dataset). The final values of *m* and *n_min_* were 0.3p and 5, respectively. The RFR was implemented using the randomForest R package version 4.7-1.1 [48].

### 2.7. Genetic Trends

To calculate genetic trends, a simple linear regression was implemented. For this, the year 1951 (the foundation of ABCCCampolina) was the genetic basis, and EBV were adjusted as follows:

(22) $${\hat{u}}_{i}^{*}={\hat{u}}_{i}-\bar{{\hat{u}}_{1951}}$$

in which ${\hat{u}}_{i}^{*}$ represents the i^th^ EBV adjusted for the basis; ${\hat{u}}_{i}$ is the EBV from the ith horse from the MTM; and $\bar{{\hat{u}}_{1951}}$ is the EBV average in 1951. The genetic trends were assessed by plotting EBV averages for each birth year and by the regression coefficient from the following linear model:

(23) $${\hat{u}}_{ij}^{*}=\beta_{0}+\beta_{1}{year}_{i}+e_{ij}$$

where ${\hat{u}}_{ij}^{*}$ is the EBV of the i^th^ horse for the j^th^ trait adjusted for the basis; $\beta_{0}$ is the intercept; $\beta_{1}$ is the regression coefficient; ${year}_{i}$ is the birth year of the i^th^ horse; and $e_{ij}$ is the random residual term. This procedure was calculated using the function lm from the stats R package [49].

### 2.8. Validation

The validation approach used in this study was the linear regression (LR) method, as proposed in [50]. The original dataset was split into training (horses born until 2010) and validation/testing (horses born in 2011, 2012, and 2013). For the validation of the MTM, we compared the EBV predicted with the complete (whole) data versus the EBV predicted with the training data only (partial). For the validation of the MLT, the EBV whole predicted via MTM was compared with the EBV predicted through MLT (ANN, SVR, or RDF) with the testing data (i.e., using all of the information only for focal animals). Accuracy (acc), bias (δ), and dispersion (b_1_) were calculated as follows:

(24) $$acc=\sqrt{cov\left({\hat{u}}_{whole},{\hat{u}}_{partial}\right)/\left(1-\bar{F}\right)\sigma_{u}^{2}}$$

(25) $$\delta =\left(\bar{{\hat{u}}_{partial}}-\bar{{\hat{u}}_{whole}}\right)/\sigma_{u}$$

(26) $$b_{1}=cov\left({\hat{u}}_{whole},{\hat{u}}_{partial}\right)/var\left({\hat{u}}_{partial}\right)$$

where ${\hat{u}}_{whole}$ is the vector of the EBV whole predicted through MTM; ${\hat{u}}_{partial}$ is the EBV partial predicted via MTM or MLT (ANN, SVR, or RDF); $\bar{F}$ is the average pedigree-inbreeding coefficient for the validation animals; $\bar{{\hat{u}}_{partial}}$ and $\bar{{\hat{u}}_{whole}}$ are the average predictions from partial and whole, respectively; and $\sigma_{u}$ is the additive genetic standard deviation. The validation was performed with in-house scripts in R [49], and to show the results, the ggplot R package [51] was used (for this and all other graphs in this study). Additionally, the correlation between whole and partial predictions (COR) and the MSE were calculated as follows:

(27) $$COR=cov\left({\hat{u}}_{whole},{\hat{u}}_{partial}\right)/\sqrt{var\left({\hat{u}}_{whole}\right)var\left({\hat{u}}_{partial}\right)}$$

(28) $$MSE=\sum_{i=1}^{n}{\left({\hat{u}}_{{whole}_{i}}-{\hat{u}}_{{partial}_{i}}\right)}^{2}/n$$

## 3. Results

### 3.1. Genetic Parameters

**Figure 2:**
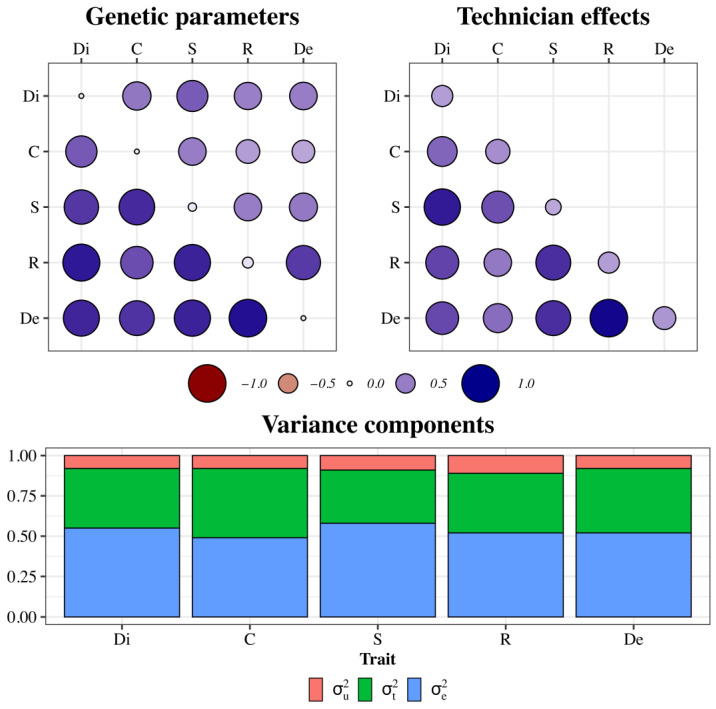
Genetic parameters—genetic correlations (above diagonal), heritability estimates (diagonal), and residual correlations (below diagonal); technician effects—proportion of phenotypic variance (diagonal) and technician correlations (below diagonal); and variance components (standardized). Abbreviations: Di = Dissociation; C = Comfort; S = Style; R = Regularity; and De = Development.

The heritability estimates ranged from 0.08 (Di, C, and De) to 0.11 (R), whereas the proportion of phenotypic variance due to technician effects ranged between 0.33 (S) and 0.43 (C) (Figure 2). The genetic correlations were all positive, varying from 0.65 (Di, C) to 0.95 (R, De), whereas the residual correlations were slightly smaller, ranging from 0.34 (De, C) to 0.78 (De, R) (Figure 2). The correlations among technician effects were positive, ranging between 0.52 (C, R) and 0.98 (R, De) (Figure 2). In addition, Table A1 shows the estimated values of the genetic and residual variance components, and Table A2 shows the estimated values of the technician variance components.

### 3.2. Genetic Trends

**Figure 3:**
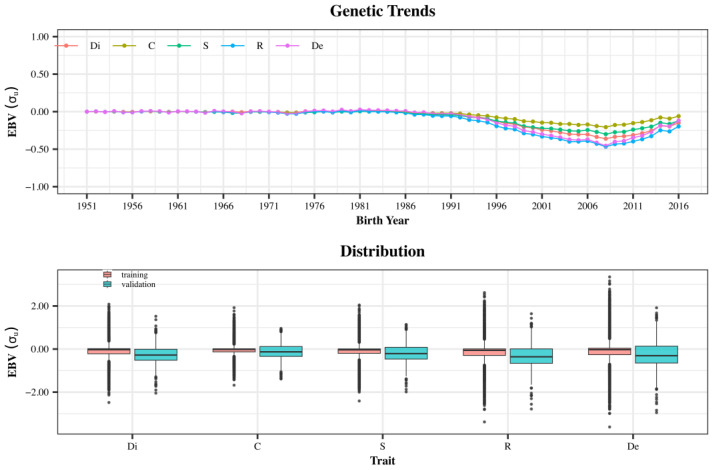
Genetic trends since breeders’ association foundation (1951) and distribution of breeding values (from multiple-trait model) for training and validation populations. Abbreviations: Di = Dissociation; C = Comfort; S = Style; R = Regularity; and De = Development.

The genetic trends were small for all studied traits (Figure 3). The regression coefficients of the EBV (predicted breeding value) on year of birth varied from −0.005 (C) to −0.011 (R). From 1951 to 1986, the trends were flat with no genetic gain. Even though the overall trend is negative, the average EBV increased from 2008 to 2016 (Figure 3). The average EBV in 2015 was slightly smaller than the average EBV in 1951 (in terms of genetic standard deviations), which is reflected in the validation animals (born in 2011, 2012, and 2013), representing a sample of the entire population (Figure 3).

### 3.3. Validation

**Figure 4:**
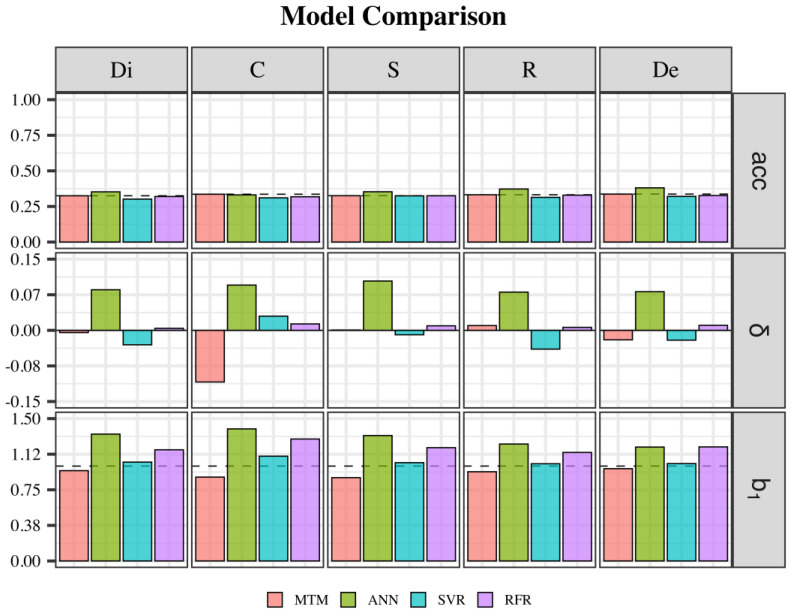
Validation statistics for each trait across all tested models. Dashed lines for acc represent the MTM acc level; for δ, it is set at its expectation (0); and for b_1_, it is set at 1 (b_1_ expectation). Abbreviations: Di = dissociation; C = comfort; S = style; R = regularity; De = development; MTM = multiple-trait model; ANN = artificial neural network; SVR = support vector regression; RFR = random forest regression; acc = accuracy; δ = bias; and b_1_ = dispersion.

Considering the MTM as the benchmark for the validation statistics, the initial values of acc (accuracy) were 0.33 for Di, S, and R and 0.34 for C and De (Figure 4). The level of bias was close to zero for all traits except for C (−0.11). For Di and S, the δ was 0.00; for R, it was 0.01; and for De, it was −0.02 (Figure 4). The dispersion bias was closer to one for Di, R, and De (varying from 0.94 to 0.97 for R and De, respectively), and it was 0.88 for C and S. When comparing the alternative methods (ANN, SVR, and RFR) to the MTM, for all of the traits, the acc was slightly higher when using an ANN (value), while SVR and RFR were slightly less accurate than the MTM (Figure 4).

The level of bias was higher for C compared to all other traits, and SVR and RFR were marginally more biased than the MTM. For all traits, the predictions from the ANN were more biased, except for C, where the MTM showed the highest level of bias. The b_1_ was higher than one in all alternative models (ANN, SVR, and RFR); however, the SVR performed better than the other MLT (Figure 4). For S, R, and De, SVR improved the b_1_ compared to the MTM, whereas for C, it was much greater than one, and for Di, the MTM was already closer to one (Figure 4). In addition, all MLTs had higher values of b_1_, which could lead to under-dispersed prediction, especially in such low-heritability traits as the ones in this study. Additional validation results are shown in Table A3.

### 3.4. Predictions

**Figure 5:**
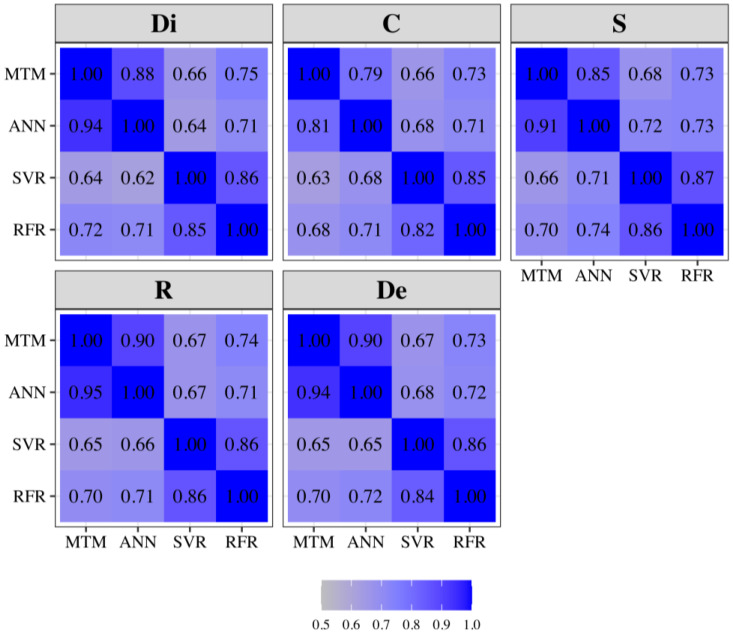
Spearman rank correlation (below diagonal) and Pearson correlation (above diagonal) of predictions across all models. Abbreviations: Di = Dissociation; C = Comfort; S = Style; R = Regularity; De = Development; MTM = Multiple-Trait Model; ANN = Artificial Neural Network; SVR = Support Vector Regression; RFR = Random Forest Regression.

The correlation between whole and partial predictions varied from 0.63 (C and S) to 0.68 (Di, R, and De) for the MTM; on the other hand, it ranged between 0.80 (C) and 0.90 (Di and De) for the ANN (Table 5). The SVR showed an overall slightly smaller correlation (ranging from 0.66 to 0.68 for C/Di and S, respectively) than the MTM, while the RFR had marginally higher values (varying between 0.72 and 0.74 for C and Di/R/De, respectively) (Table 5). The MSE was smaller for the ANN than the MTM, while it was comparable between the SVR/RFR and the MTM. For all MLTs, the MSE was smaller or equal to the one from the MTM, except for Di from the SVR model, which had a slightly higher value (Table 5). The mean, minimum, and maximum showed that the predictions were skewed to the left for all MLTs, with much more shrunken values than the MTM (Table 5). The standard deviation, however, was similar within traits across methods. Additionally, the Spearman rank correlation and the Pearson correlation among predictions for each trait across all models are presented in Figure 5.

## 4. Discussion

Estimates of the additive genetic variance found in this study suggest that the Campolina population could be selected for gait scores, although with a moderate genetic gain per year. Selection could be conducted based on the trait with the highest heritability (regularity) since the genetic correlations were all positive from moderate to high magnitude, meaning selection for any of these gait scores will cause improvements in all the others by the correlated response. If the population is undergoing selection, young animals will have a higher or lower EBV (predicted breeding value) average depending on whether the selection is for a higher or lower EBV. In our study, the validation animals had a slightly lower average EBV (compared to the population average), which can be explained by the behavior of the genetic trends marginally decreasing from 1991 to 2008 and recovering from 2009 to 2016. The selection of Campolina horse is based on show and phenotypic records. Gait scores were introduced in the registration process by the ABCCCampolina in the 1990s. Before that, the genetic trend is flat, which reflects the lack of selection. After that, the trend is negative, possibly due to the wrong choice of stallions or due to the bottleneck the population experienced between 1989 and 1996 [52,53]. Those results support the study by Bussiman et al. [11], who showed that some genetic progress exists for morphological traits in Campolina horses; however, gait showed no genetic gain. Bussiman et al. [11] reported a heritability for gait of 0.07, and Bussiman et al. [13] found a heritability of 0.16 while using a different definition of contemporary groups (and criteria to clean the dataset) for the same trait in the same population. Our heritability estimates support those findings, which suggest that gait (and different gait attributes) in Campolina horses has low heritability.

The technician effect may be responsible for a larger proportion of phenotypic variance, ranging from 0.13 [13] to 0.60 [11], while in our study, it varied from 0.33 to 0.43, evidencing the high subjectivity in those visual scores. Along with the subjectivity, functional traits in horses are commonly highly affected by environmental forces [54,55]; such effects could be riders [12,56,57], appraisers/technicians [8,11], competitions/events [56,58], or even other horses if the trait comprises competition records [55,56,58,59].

The challenge, then, is to overcome such subjectivity and environmental effects. For Campolina horses, the problem was first addressed by Bussiman et al. [11], who modeled technicians as an uncorrelated random effect for “Gait total Score” (GtS) and stated that frequent training could help to reduce the amount of phenotypic variance due to this effect. GtS corresponds to the sum of dissociation, comfort, style, regularity, and development [11], and since the same technician assigns all the scores, one should expect high technician correlations.

Our findings suggest that the technician effect is not the same for all traits, implying that the scores might be assigned according to the gait type. Different technicians may have different preconceptions regarding the dissociation, comfort, style, regularity, and development values depending on whether the horse performs MP or MB. The high correlations between dissociation and style (0.91), and between regularity and development (0.98) emphasize this reasoning. Style is related to how elegant the horses’ movements are perceived to be; regularity values reflect the horses’ ability to maintain the same gait for long periods of time; development is related to the number of steps; and dissociation is associated with limb coordination. MP has a higher step frequency [11] and a higher dissociation than MB [60,61]. The perception of “beauty” (or elegance) might also be related with regional differences since different regions of Brazil tend to prefer MP over MB, and vice versa.

To the best of our knowledge, this is the first attempt to use common validation methods applied in animal breeding to assess predictions from MLTs (machine learning techniques). Commonly, for regression purposes, the Pearson correlation is the accuracy measurement used in MLTs [62]; however, in an animal breeding context, Legarra and Reverter [50] showed that the expected value of the correlation between subsequent genetic evaluations is equal to the ratio between their respective accuracies. Moreover, the LR method deals with the models’ ability to rank a selected set of focal/validation animals [50,63].

Shahinfar et al. [64], working with dairy cattle, applied an ANN to predict the EBV for milk production and reported a correlation of 0.90 between the EBV from the ANN with the EBV from mixed models. Ghotbaldini et al. [65] and Pour Hamidi et al. [66] found a higher determination coefficient (R^2^) for ANN predictions. However, these authors did not report R^2^ values for mixed model predictions. Our results support these findings since we found that ANN predictions had a higher correlation with the EBV (predicted breeding value) from the MTM (multiple-trait model). However, the ANN predictions were more biased in our study. This can be related to the fact all MLTs used the EBV to train the model and estimate the loss function. In this sense, the MLTs had extra information since the MTM predicts the breeding value from the phenotype. On the other hand, training MLTs with the phenotype would lead to phenotype prediction.

In an animal breeding context, bias is usually split into level bias and dispersion bias [50]. Level bias is usually related to the response to selection and can be ignored when there is no re-ranking [24]. Dispersion bias (or simply dispersion) can affect the genetic gain because if predictions are over-dispersed, too many young animals are selected (and if they are under-dispersed, too few young animals are selected), which can hamper the genetic trend [67]. The rank of the focal animals changed across all methods, and the highest rank correlation was found between the ANN and MTM (followed by RFR and SVR) across all traits. Predictions from the ANN (followed by RFR) showed the highest under-dispersion. If selection decisions are based on the EBV from MLTs, one should consider that re-ranking may impact genetic trends, at least at the beginning.

Zhao et al. [68] applied SVR to genomic EBV prediction in pigs and maize and reported similar accuracy between SVR and genomic mixed models. Moser et al. [69] showed that SVR had dispersion closer to one than traditional methods for dairy bulls, yet the accuracy was similar across all tested methods. In our study, SVR had comparable accuracy to the MTM, was less biased, and had better dispersion coefficients (except for C). These results support the need to investigate different regression kernels for different traits [70,71]. Sandhu et al. [72], working with wheat, conducted an extensive comparison of different MLTs for genomic EBV prediction. RFR performed better than SVM and traditional mixed models in all scenarios. The same authors found that a more straightforward ANN configuration (multilayer perceptron) was as accurate as the RFR.

The prediction from linear mixed models requires enough data [16]. In our study, it is possible that the reduced number of phenotypic observations caused a reduced prediction accuracy from the MTM. Moreover, the dispersion coefficient being smaller than one for all traits can also be explained by the reduced number of phenotypic records. In all models, when the validation animals were used to train the models, the predictions had accuracy and dispersion equal to 1.00 and bias equal to 0.00.

It is possible that the MTM predictions did not have enough theoretical accuracy to train alternative models. MLTs might be trained with the “noise” associated with each animal’s EBV if that is the case. That could explain the bias and dispersion increase for some traits when using MLTs. The reduced information and data structure can explain the lack of theoretical accuracy. The maximum values of theoretical accuracy (from MTM) were 0.56 (dissociation), 0.56 (comfort), 0.57 (style), 0.58 (regularity), and 0.57 (development). Another possibility is that the OLS solutions for the fixed effects were not well estimated, carrying some extra level of uncertainty to the MLT.

However, the MLT somehow involves “multistep prediction” since we need to have the breeding value to train the model, which can be overcome by using the adjusted phenotype to train the model. It is possible that the prediction from the MLT can be interpreted as the EBV by using the adjusted phenotype, but this remains unclear. Generally, producers only use the EBV to rank the animals, mating all individuals surpassing a given selection threshold. Another way of validating our predictions could be the percentage of animals selected in common with the MTM for a given fixed selection threshold (say, the top 10%); however, this was not within the scope of this study since the rank correlations were already presented. Machine learning algorithms are very powerful prediction tools, but interpretation and inference are compromised since, in most cases, models are so complex that it is nearly impossible to evaluate each parameter. On the other hand, the prediction accuracy of traditional methods, such as linear mixed models, is often higher than that of MLT, whose architecture is “too simplistic”.

Even though the MLTs were effective in reducing the MSE, the predictions for all tested alternative models were skewed to the left and more shrunken than those of the MTM. The ANN predictions were completely truncated at −0.17, while RFR had the most similar amplitude compared to the MTM. This could be a result of the activation function in the output layer and since a truncated distribution might have a smaller standard deviation; this could also explain the higher correlation found for the ANN. Despite the differences in scale, the variance of the predictions was similar for all models, while it was smaller for all MLTs.

It was not the purpose of this study to fine-tune the MLTs; instead, we aimed to show their potential for breeding value prediction for traits with low heritability and a high degree of subjectivity. Montesinos et al. [62] argued that model tuning is always needed to find the most accurate model. Tuning models’ hyperparameters would also help to avoid overfitting [30,62]. Alves et al. [28] suggested using a genetic algorithm to find the best model architecture, and Hastie et al. [73] argued that tuning should be based on the prediction error. Even though we did not further explore the fine-tuning of our models, we achieved good generalization, and the MLT predictions were comparable to those of the MTM, showing the potential of machine learning for these traits.

The computing cost was not directly measured in our study. The time to obtain predictions highly depends on the amount of data and computing resources available. For the MTM, all analyses were concluded in less than one day, while for MLTs (including the time to calculate OLS solutions), carrying out training and predictions lasted two hours. Finally, if predictions are needed for new animals until they are included in official evaluation runs, we recommend using previous solutions to train an SVR to predict the EBV for those animals. This model had accuracy, bias, and dispersion comparable to the MTM for all studied traits.

Furthermore, we are not advocating for the replacement of traditional mixed models. All of the alternative models presented in this study should be cautiously assessed since more research is needed, mainly to implement more robust tuning methods and evaluate the genetic gain if machine learning predictions are used for selection.

## 5. Conclusions

For the population in this study, prediction via machine learning techniques is a feasible alternative. However, the estimated breeding values for young animals will be slightly biased and over-dispersed, which can harm the genetic trend, especially for low heritability traits. To overcome this problem, we recommend a more comprehensive tuning method for artificial neural networks, support vector regression, and random forest regression. Support vector regression performed better than all other alternative models tested in terms of dispersion, while the artificial neural network had the highest prediction accuracy. However, special attention should be paid to the statistical model and the phenotypic dataset for the least square procedure before model training; this could improve the performance of all machine learning techniques and be an interesting field of future research.

## Figures and Tables

**Table 1:** The descriptive statistics of the raw dataset.

Trait	Mean	SD ^1^	Min	Max	NF ^2^	NM ^3^	CG ^4^	Stud ^5^	Tec ^6^
Di	30.73	3.70	17	51	4162	1706	582	853	46
C	47.66	5.10	26	61	4178	1713	596	857	46
S	30.34	5.36	1	52	4178	1713	596	857	46
R	22.76	2.54	15	37	4178	1713	596	857	46
De	22.82	2.57	15	36	4178	1713	596	857	46

^1^ Standard deviation; ^2^ number of females; ^3^ number of males; ^4^ number of contemporary groups; ^5^ number of studs (same as herd for cattle reader); and ^6^ number of technicians. Abbreviations: Di = Dissociation; C = Comfort; S = Style; R = Regularity; and De = Development.

**Table 2:** Descriptive statistics of the clean dataset used for variance component estimation.

Trait	Mean	SD ^1^	Min	Max	NF ^2^	NM ^3^	CG ^4^	Stud ^5^	Tec ^6^
Di	30.41	3.79	21	41	2542	1179	139	117	28
C	47.63	4.89	33	61	3567	1456	203	134	28
S	30.60	3.75	21	41	2728	1142	155	116	28
R	22.96	2.62	16	30	2674	1091	150	147	28
De	23.00	2.56	16	30	2731	1017	154	179	28

^1^ Standard deviation; ^2^ number of females; ^3^ number of males, ^4^ number of contemporary groups; ^5^ number of studs (same as herd for the cattle reader); and ^6^ number of technicians. Abbreviations: Di = Dissociation; C = Comfort; S = Style; R = Regularity; and De = Development.

**Table 3:** Number of phenotypic records (diagonal bold) and number of animals with records for every two traits (above diagonal) in clean dataset.

Trait	Trait				
	Di	C	S	R	De
Di	**3721**	3373	3065	2670	2669
C		**5023**	3494	3319	3334
S			**3870**	2728	2756
R				**3765**	3251
De					**3748**

Abbreviations: Di = Dissociation; C = Comfort; S = Style; R = Regularity; and De = Development.

**Table 4:** Descriptive statistics of the adjusted phenotypes and ordinary least squares solutions for each of the traits.

Model Effect	Statistic	Trait				
		Di	C	S	R	De
Adjusted phenotype	Mean	−0.05	0.00	0.00	0.00	0.00
	Min	−33.24	−21.34	−27.37	−8.58	−7.50
	Max	20.84	17.65	19.84	14.96	11.58
	SD	2.85	3.63	3.06	1.92	1.97
CG	Mean	9.49	−0.12	−1.17	−0.23	−0.42
	Min	−45.33	−45.35	−98.01	−18.15	−16.83
	Max	38.19	53.87	31.28	22.68	27.15
	SD	15.33	3.04	5.37	1.34	1.31
Stud	Mean	23.87	51.83	34.39	24.39	24.82
	Min	−8.03	0.00	−45.57	0.00	0.00
	Max	49.34	66.89	66.84	33.65	39.44
	SD	15.24	4.24	3.91	1.74	1.77
Age (linear)	Mean	−5.37	−7.09	−8.22	−2.60	−2.75
	Min	−29.74	−39.26	−45.57	−14.39	−15.23
	Max	−2.53	−3.35	−3.88	−1.23	−1.30
	SD	2.41	3.18	3.69	1.17	1.23
Age (quadratic)	Mean	2.66	3.04	5.34	1.20	1.17
	Min	0.49	0.56	0.99	0.22	0.22
	Max	68.02	77.68	136.53	30.63	29.93
	SD	3.71	4.23	7.44	1.67	1.63

Abbreviations: CG = contemporary group; Min = minimum value; Max = maximum value; and SD = standard deviation.

**Table 5:** Descriptive statistics of the predictions (EBV) for each trait from different models.

Statistic	Model	Trait				
		Di	C	S	R	De
COR	MTM	0.68	0.63	0.63	0.68	0.68
	ANN	0.90	0.80	0.85	0.89	0.90
	SVR	0.66	0.66	0.68	0.67	0.67
	RFR	0.74	0.72	0.73	0.74	0.74
MSE	MTM	0.13	0.28	0.17	0.09	0.07
	ANN	0.07	0.20	0.10	0.04	0.03
	SVR	0.14	0.25	0.15	0.09	0.07
	RFR	0.12	0.22	0.13	0.08	0.06
Mean	MTM	−0.32	−0.34	−0.23	−0.24	−0.16
	ANN	0.10	0.15	0.12	0.09	0.07
	SVR	−0.03	0.04	−0.01	−0.04	−0.02
	RFR	0.01	0.02	0.01	0.00	0.01
Min	MTM	−2.29	−2.45	−2.27	−1.97	−1.51
	ANN	−0.17	−0.17	−0.17	−0.17	−0.17
	SVR	−0.93	−1.20	−0.97	−0.77	−0.65
	RFR	−1.28	−1.50	−1.34	−1.18	−0.93
Max	MTM	0.86	1.75	1.19	0.75	0.78
	ANN	1.13	1.29	1.30	1.25	1.12
	SVR	1.23	1.66	1.33	1.13	1.08
	RFR	1.08	1.44	1.20	0.92	0.80
SD	MTM	0.36	0.47	0.38	0.30	0.25
	ANN	0.33	0.39	0.35	0.31	0.27
	SVR	0.32	0.39	0.35	0.27	0.24
	RFR	0.32	0.37	0.32	0.26	0.22

Abbreviations: Di = Dissociation; C = Comfort; S = Style; R = Regularity; De = Development; MTM = Multiple-Trait Model; ANN = Artificial Neural Network; SVR = Support Vector Regression; RFR = Random Forest Regression; COR = Correlation Between Whole and Partial Predictions; MSE = Mean Squared Error; Min = Minimum; Max = Maximum; and SD = Standard Deviation.

## Data Availability

The dataset presented in this study is not readily available because the raw dataset is property of the Brazilian Campolina Horse Breeders Association (ABCCCampolina), and this information is commercially sensitive. Requests to access the datasets should be directed to ABCCCampolina (https://www.campolina.org.br/ accessed on 10 August 2024).

## Appendix A

The estimated variance components are illustrated in Table A1, and the technician effects (as a proportion of phenotypic variance, along with correlations) are shown in Table A2. Since the variance components were estimated with EM-REML, no standard error is presented.

**Table A1:** Estimated genetic parameters—heritability (diagonal bold), genetic correlations (above diagonal), and residual correlations (below diagonal).

Trait	Trait				
	Di	C	S	R	De
Di	**0.08**	0.65	0.79	0.92	0.87
C	0.53	**0.08**	0.85	0.70	0.81
S	0.64	0.50	**0.09**	0.88	0.88
R	0.49	0.37	0.50	**0.11**	0.95
De	0.50	0.34	0.52	0.78	**0.08**

Abbreviations: Di = Dissociation; C = Comfort; S = Style; R = Regularity; and De = Development.

**Table A2:** Estimated technician parameters—proportion of phenotypic variance explained by technician (diagonal bold) and technician correlations (above diagonal).

Trait	Trait				
	Di	C	S	R	De
Di	**0.37**	0.60	0.91	0.74	0.72
C		**0.43**	0.69	0.52	0.57
S			**0.33**	0.83	0.83
R				**0.37**	0.98
De					**0.40**

Abbreviations: Di = Dissociation; C = Comfort; S = Style; R = Regularity; and De = Development.

## Appendix B

The validation statistics (accuracy, bias, and dispersion) for all models across all traits are presented in Table A3.

**Table A3:** Validation statistics for all tested models across different traits.

Statistic	Model	Trait				
		Di	C	S	R	De
Accuracy	MTM	0.33	0.34	0.33	0.33	0.34
	ANN	0.37	0.34	0.35	0.39	0.39
	SVR	0.30	0.31	0.32	0.31	0.32
	RFR	0.32	0.32	0.33	0.33	0.33
Bias	MTM	0.00	−0.11	0.00	0.01	−0.02
	ANN	0.11	0.11	0.09	0.10	0.08
	SVR	−0.03	0.03	−0.01	−0.04	−0.02
	RFR	0.01	0.01	0.01	0.01	0.01
Dispersion	MTM	0.95	0.88	0.88	0.94	0.97
	ANN	1.21	1.34	1.39	1.19	1.25
	SVR	1.04	1.10	1.04	1.02	1.03
	RFR	1.17	1.29	1.19	1.14	1.20

Abbreviations: Di = dissociation; C = comfort; S = style; R = regularity; De = development; MTM = multiple-trait model; ANN = artificial neural network; SVR = support vector regression; and RFR = random forest regression.
